# Inhibitory Activities of Phenolic Compounds Isolated from *Adina rubella* Leaves Against 5α-Reductase Associated with Benign Prostatic Hypertrophy

**DOI:** 10.3390/molecules21070887

**Published:** 2016-07-07

**Authors:** Jun Yin, Jun Hyeok Heo, Yoon Jeong Hwang, Thi Tam Le, Min Won Lee

**Affiliations:** Laboratory of Pharmacognosy and Natural Product Derived Medicine, College of Pharmacy, Chung-Ang University, Seoul 156-756, Korea; yinjun89@naver.com (J.Y.); fifasthur@naver.com (J.H.H.); g_intention@naver.com (Y.J.H.); letam18692@gmail.com (T.T.L.)

**Keywords:** *Adina rubella* Hance, anti-oxidative, anti-inflammatory, 5α-reductase, benign prostatic hypertrophy

## Abstract

*Adina rubella* Hance (AR), a plant native to Korea, has been used as traditional medicine for dysentery, eczema, intoxication, and external hemorrhages. Previous phytochemical studies of AR have reported several components, including terpenoids, phenolics, and alkaloids. The current study evaluated the anti-oxidative and anti-inflammatory activities and 5α-reductase inhibition of isolated compounds of AR leaves to find a potential therapeutic agent for benign prostatic hypertrophy (BPH). Repeated chromatographic isolation of an 80% acetone extract of AR leaves yielded seven phenolic compounds: caffeic acid (**1**), chlorogenic acid (**2**), methyl chlorogenate (**3**), quercetin-3-rutinoside (**4**), kaempferol-3-*O*-α-l-rhamnopyranosyl-(1→6)-β-d-glucopyranoside (**5**), hyperoside (**6**), and grandifloroside (**7**). Compound **7** is a novel compound in AR. Caffeoyl derivatives **1**–**3** and **7** showed good anti-oxidative activities. In particular, caffeic acid (**1**) and grandifloroside (**7**) showed potent anti-inflammatory activities, and **7** also exhibited potent inhibitory activity against TNF-α and 5α-reductase. Our results show that the extract and grandifloroside (**7**) from leaves of AR might be developed as a source of potent anti-oxidative and anti-inflammatory agents and therapeutic agent for BPH.

## 1. Introduction

*Adina rubella* (AR), a plant that is native to Korea and endemic to Mount Hanlla on Jeju Island, is a traditional Korean medicine used for the treatment of dysentery, eczema, intoxication, diarrhea, odontalgia, and external hemorrhages. Previous phytochemical studies of AR have mainly focused on the roots and have yielded several triterpenoid saponins, alkaloids, and some phenolics [1,2,3,4]. There are reports on pharmacological investigations of AR that showed inhibitory activity on production of nitric oxide and antibacterial, antiviral and antitumor activities [5,6,7].

Benign prostatic hypertrophy (BPH) is an overgrowth of prostate tissue caused by androgen-dependent tissue remodeling, prostate cancer, and proliferation of prostate tissue [8,9,10]. Enlargement of prostate tissue can cause lower urinary tract symptoms (LUTS), which hinder urination [11].

The microsomal enzyme 5α-reductase catalyzes the NADPH-dependent reduction of testosterone as a Δ^4^-3-ketosteroid to dihydrotestosterone (DHT) as a 3-ketosteroid [12,13,14]. The transport of DHT bind to androgen receptor and the complex one results in a cascade of events necessary for the formation of signaling factors that regulate cellular growth to lead BPH [15]. Thus, 5α-reductase inhibitor may be a way to treat BPH. This paper describes the isolation of phenolic compounds from the leaves of AR and their anti-oxidative, anti-inflammatory, and 5α-reductase inhibition activities associated with BPH. It was reported that some phenolic compounds and plant extracts showed inhibitory of inflammatory, cytokines, and IgE productions. However, studies of their inhibitory activity on 5α-reductase are rare [16,17,18,19,20,21].

## 2. Results and Discussion

**Figure 1:**
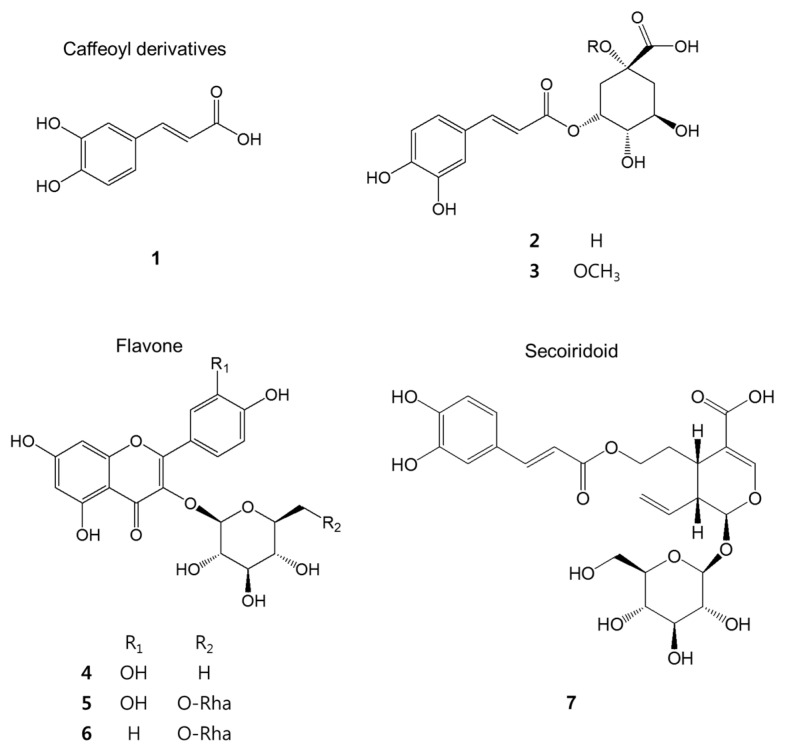
The chemical structure of the isolated compounds.

Repeated chromatographic fractionation of an 80% acetone extract of AR leaves yielded seven phenolic compounds, structurally identified as three caffeoyl derivatives: caffeic acid (**1**) [22], chlorogenic acid (**2**) [23], methyl chlorogenate (**3**) [24]; three flavonoids: quercetin-3-*O*-rutinoside (**4**) [25], kaempferol-3-*O*-α-l-rhamnopyranosyl-(1→6)-β-d-glucopyranoside (**5**) [26], hyperoside (**6**) [27]; and one secoiridoid, grandifloroside (**7**) [28] by comparison of their spectral data with those of described in literature or direct comparison with authentic samples (Sigma-Aldrich, St. Louis, MO, USA) (Figure 1) Grandifloroside (**7**) is the first secoiridoid isolated from AR.

Reducing oxidative stress is related to anti-inflammatory effect [29]. Free radicals play a role in early carcinogenesis and BPH might be considered a premalignant condition [30]. There is a strong correlation between inflammation and the pre-cancerous lesions [31], and inducible nitric oxide synthase has been detected in a BPH patient [32], indicating a possible role for NO in the pathogenesis of BPH. Thus increased anti-oxidant and anti-inflammatory activities could be helpful to treat BPH.

To assess the anti-oxidative activity of the compounds isolated from AR, their DPPH radical and NBT superoxide scavenging activities were measured. All of the compounds **1**–**7** showed anti-oxidative activity except **6**. In particular, methyl chlorogenate (**3**) and grandifloroside (**7**) showed potent DPPH radical scavenging activities compared with the positive control, l-ascorbic acid (IC_50_ = 23.88 ± 0.76 µM). These compounds also showed potent superoxide scavenging activities, except **6** and compared with positive control, allopurinol (IC_50_ = 4.57 ± 0.73 µM). (Table 1) The caffeoyl derivatives displayed stronger anti-oxidative activities than flavonoids. Caffeic acid (**1**) (IC_50_ = 23.98 ± 1.52) and grandifloroside (**7**) (IC_50_ = 30.12 ± 1.02) showed strong inhibition of NO production compared with the positive control, l-NMMA (IC_50_ = 29.12 ± 1.98 μM). (Table 1) Also, the anti-oxidative and anti-inflammatory activities of fractions including extract from AR were measured (Table 2).

Interleukin (IL)-1β and IL-6 as pro-inflammatory cytokines are significantly increased in BPH patients [33,34], The presence of Il-6 in human prostatic carcinomas and BPH has been reported [35,36,37]. IL-6 plays a key role as a growth factor for normal prostatic tissue as well as for prostate cancer cells [38,39]. Thus, IL-6 cytokine may lead to hyperplasia of prostate. The IL-1 family includes two types of ligands, IL-1β and IL-1α [40], and the IL-1 family has been detected in prostate cancer [41]. Also, IL-1β and tumor necrosis factor-alpha (TNF-α), which is a tumoral promoter cytokine, also are important in the treatment of chronic prostatitis patients [42]. TNF-α cytokine has anti-tumor activity and has a variety of cellular responses including apoptotic and proliferation activities [43,44]. Thus, TNF-α cytokine may reduce the tissue of hyperplasia prostate and decrease of the cytokines of IL-1β, IL-6 and TNF-α could be helping to treat BPH.

**Figure 2:**
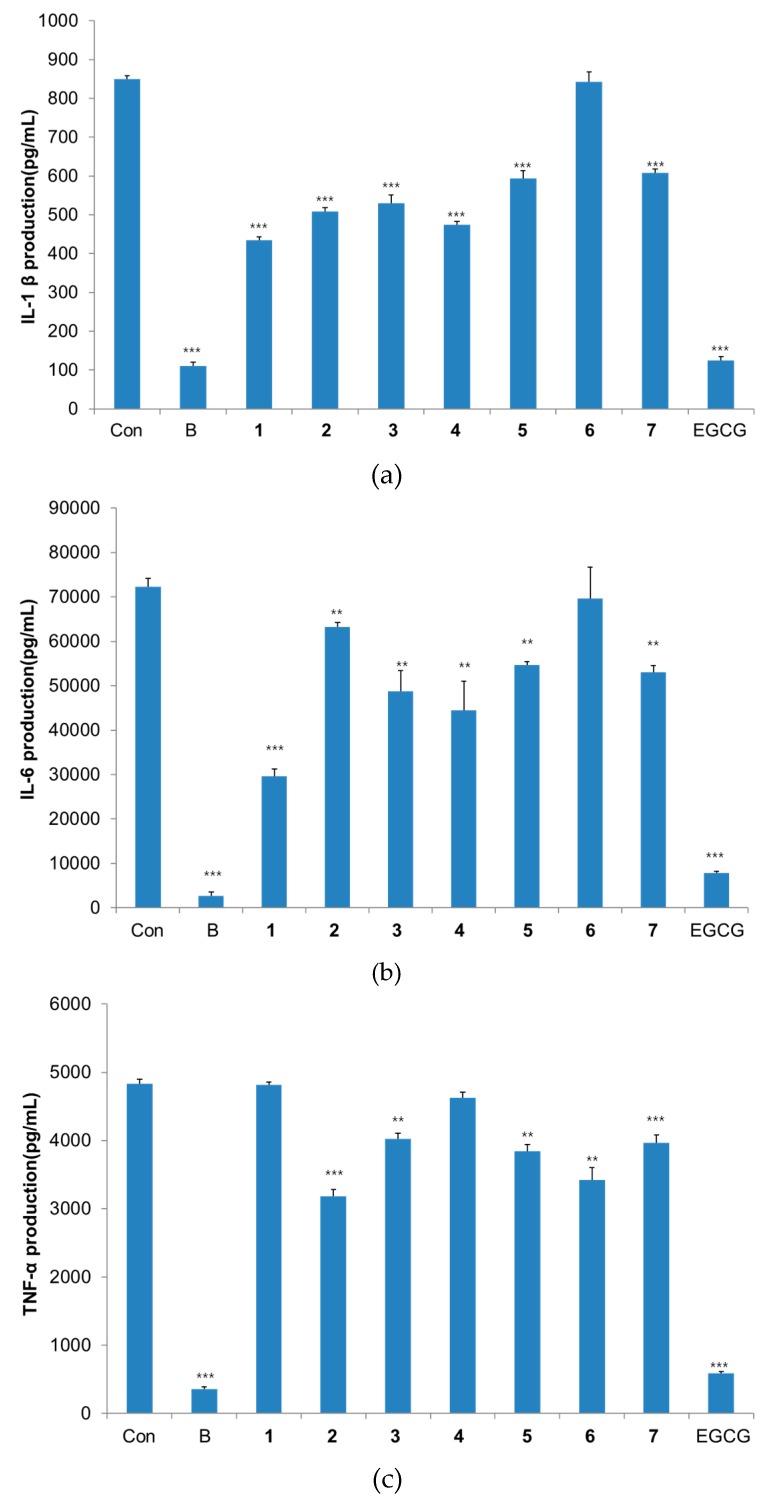
Inhibitory activities on cytokines production in THP-1 macrophage cells. Results are expressed as mean ± S.D. (*n* = 3). ** *p* < 0.01, *** *p* < 0.001 versus control group. Con, negative control group; B, blank group (normal); EGCG, positive control. (**a**) IL-6 production inhibitory activity; (**b**) TNF-α production inhibitory activity; (**c**) IL-1β production inhibitory activity.

To examine the inhibitory effect of the phenolic compounds on factors associated with BPH, the inhibitory activity on 5α-reductase and cytokines (IL-6, IL-1β, TNF-α) production in LPS-stimulated THP-1 cells were evaluated. Compounds **1**–**4** strongly inhibited IL-1 β cytokine and **1**, **3**–**5** displayed potent IL-6 cytokine inhibition activities. And **2**, **5**–**7** inhibited TNF-α cytokine strongly (Figure 2).

**Figure 3:**
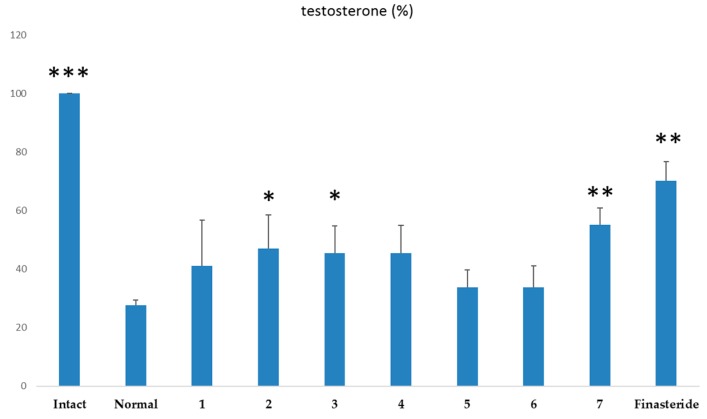
Inhibitory activities on 5α-reductase with testosterone as substrate. Values were expressed as mean ± S.D of triplicate measurements (*n* = 3) at four independent experiments. * *p* < 0.05, ** *p* < 0.01, *** *p* < 0.001 versus control group.

The 5α-reductase inhibitory activity of the compounds was measured. In the present work, compounds **2**, **3** and **7** significantly increased testosterone content compared with the positive control, finasteride, which is widely used as a BPH therapeutic drug via the inhibition of 5α-reductase (Figure 3). It is reported that chlorogenic acid (**2**) showed inhibitory effects on the 5α-reductase enzyme [16]. In our experiment, **2** displayed potent inhibition of 5α-reductase, close to 50% as determined by the concentration of testosterone and this is the first time the inhibitory activities on 5α-reductase of these isolated compounds is reported, except for chlorogenic acid (**2**).

Hiipakka et al. reported that the *ortho*-dihydroxyphenol moiety of phenolic compounds has inhibitory activities on 5α-reductase [45]. Compounds **2**, **3** and **7** which are kinds of caffeoyl derivatives might show significant 5α-reductase inhibitory activity.

Grandifloroside (**7**) has similar structure as oleuropein, which is a caffeoyl iridoid glycoside and was reported to play an antiproliferative effect on prostate cell lines including BPH-1 (non-malignant prostate cell) and LNCaP and DU145 (prostate cancer cell lines) [46]. This may explain why **7** has the most beneficial activity on BPH among the phenolic compounds from AR.

## 3. Materials and Methods

### 3.1. General Procedures

Column chromatography was performed using Sephadex LH-20 (10–25 μm; GE Healthcare Bio-Science AB, Uppsala, Sweden), MCI-gel CHP 20P (75–150 μm; Mitsubishi Chemical, Tokyo, Japan), Toyopearl HW-40F (30–60 μm; Tosoh Corp., Tokyo, Japan), and ODS-B gel (40–60 μm; Daiso, Osaka, Japan). ODS-B gel was also used as a stationary phase for high-performance liquid chromatography (HPLC, Waters, coastal, CT, USA). Thin layer chromatography (TLC) was carried out using a pre-coated silica gel 60 F_254_ plate (Merck, Darmastadt, Germany) with chloroform, methanol, and water (70:30:4 and 80:20:2, volume ratio). Spots were detected under ultraviolet (UV, Waters, coastal, CT, USA) radiation (254 nm) by spraying with FeCl_3_ and 10% H_2_SO_4_ or anisaldehyde-H_2_SO_4_ followed by heating. The chemical structures were elucidated by several instrumental analyses. One-dimensional nuclear magnetic resonance (1D-NMR) included ^1^H-(300 or 600 MHz), (NMR; Varian, Palo Alto, CA, USA) and ^13^C-(150 MHz) NMR. (NMR; Varian, Palo Alto, CA, USA) spectra. Two-dimensional (2D)-NMR included proton-proton correlation spectroscopy (^1^H^1^H-COSY) and heteronuclear single quantum coherence (HSQC) spectra. Heteronuclear multiple bond coherence (HMBC) experiments were recorded with Gemini 2000 and VNS instruments (Varian, Palo Alto, CA, USA) at the center for research facilities of Chung-Ang University using an internal tetramethylsilane (TMS) standard. High-resolution fast atom bombardment mass spectra (HR-FAB-MS) were recorded with JMS-600W and JMS-700 instruments (JEOL, Tokyo, Japan) at the National Center for Inter-University Research facilities at Seoul National University. A HPLC system including a model 600 pump (Waters, coastal, CT, USA), model 717 plus auto-sampler (Waters, coastal, CT, USA), model 486 (280 nm) detector (Waters, coastal, CT, USA), and a Kromasil C^18^ HPLC column (C^18^-HL 5 μm, 250 mm × 4.6 mm, Varian) were used for quantitative surveys of the testosterone content.

### 3.2. Plant Material

Fresh leaves of AR were gathered from the Hanlla Arboretum on Jeju Island, South Korea, during July 2012. The identity of the material was confirmed by Seho Jeong (Hanlla Arboretum, Jeju Island, Korea). The leaves were dried at room temperature for 3 days. A voucher specimen was deposited at the herbarium of the College of Pharmacy, Chung-Ang University.

### 3.3. Cell Culture

Murine macrophage Raw 264.7 cells were purchased from the Korean Cell Line Bank (Seoul, Korea). These cells were grown at 37 °C in a humidified atmosphere (5% CO_2_) in Dulbecco′s Modified Eagle′s Medium (DMEM; Sigma-Aldrich, St. Louis, MO, USA) containing 10% fetal bovine serum (FBS), 100 IU/mL penicillin G, and 100 mg/mL streptomycin (Gibco BRL, Grand Island, NY, USA) [47], and were used after cell counting with a hemocytometer. THP-1 human monocytic leukemia cells purchased from the Korean Cell Line Bank were grown at 37 °C in a humidified atmosphere (5% CO_2_) in RPMI 1640 medium (Sigma-Aldrich) containing 10% FBS and 100 IU/mL penicillin G (Thermo Fisher Scientific Korea Ltd., Seoul, Korea), and were used after cell counting with a hemocytometer.

### 3.4. Extraction and Isolation

AR leaves (1.2 kg) were extracted with 80% acetone at room temperature for 2 days. A concentration process that removed the acetone under vacuum yielded 310 g of extract. After acetone evaporation, the extract was suspended with water and the aqueous solution was filtered through Celite 545 (Duksan Pure Chemicals Co., Ltd, Seoul, Korea). The filtrate was applied to a Sephadex LH-20 column (2000 g, 10 cm × 120 cm) and eluted using a gradient solvent system of H_2_O-methanol (MeOH) (from 100:0 to 0:100) to yield 10 fractions (AR-1 to 10 in order of elution).

Repeated column chromatography of fraction AR-7 (3.21 g) using a MCI gel column (400 g, 3 cm × 50 cm) with a gradient solvent system of H_2_O–MeOH (from 100:0 to 0:100) yielded three sub-fractions (AR-7.1 to 7.3). Fraction AR-7.1 (2 g) was applied to an ODS gel column (250 g, 3 cm × 50 cm) with a gradient solvent system of H_2_O–MeOH (from 80:20 to 0:100) and yielded caffeic acid (**1**; 520 mg).

Repeated column chromatography of fraction AR-5 (18.08 g) using a MCI gel column (50 μm, 18 g, 3 cm × 50 cm) with a gradient solvent system of H_2_O–MeOH (from 100:0 to 0:100) yielded seven sub-fractions (AR-5.1 to 5.7). Sub-fraction AR-5.1 (9.19 g) was subjected to ODS gel column chromatography (50 μm, 150 g, 3 cm × 50 cm) with a gradient solvent system of H_2_O–MeOH (from 100:0 to 0:100) to give chlorogenic acid (**2**; 501 mg) and methyl chlorogenate (**3**; 300 mg). The sub-fraction of AR-5.4 (720 mg) was applied to a MCI gel column (50 μm, 400 g, 3 cm × 50 cm) with a H_2_O–MeOH gradient (from 100:0 to 0:100) and an ODS gel column (50 μm, 250 g, 3 cm × 50 cm) with H_2_O–MeOH gradient (from 90:10 to 0:100) to yield quercetin-3-rutinoside (**5**; 140 mg) and kaempferol-3-*O*-α-l-rhamnopyranosyl-(1→6)-β-d-glucopyranoside (**6**; 142 mg).

Repeated column chromatography of fraction AR-6 (8.72 g) using a gradient solvent system of H_2_O–MeOH (from 100:0 to 0:100) yielded six sub-fractions (AR-6.1 to 6.6). The AS-6.5 sub-fraction was partitioned on an ODS gel column (50 μm, 250 g, 3 cm × 50 cm) using a H_2_O–MeOH gradient (from 100:0 to 0:100) to yield grandifloroside (**7**; 50 mg).

Repeated column chromatography of fraction AR-8 (5.68 g) using a H_2_O–MeOH solvent gradient (from 100:0 to 0:100) yielded three sub-fractions (AR-8.1 to 8.3). The AR-8.2 sub-fraction (350 mg) was passed through an ODS gel column (50 μm, 150 g, 3 cm × 50 cm) with a H_2_O–MeOH gradient solvent system (from 100:0 to 0:100) to give hyperoside (**4**; 100 mg).

### 3.5. Antioxidative Activity

To assess the antioxidative activity of compounds isolated from the leaves of AR, 1,1-diphenyl-2-picryl-hydrazyl (DPPH) radical scavenging activity and nitrotetrazolium blue chloride (NBT) superoxide scavenging activity were measured.

#### 3.5.1. Measurement of DPPH Radical Scavenging Activity

Antioxidant activity was evaluated on the basis of the scavenging activity of the stable DPPH free radical (Sigma-Aldrich). Twenty microliters of each sample (1000, 500, 250, 125 mM) in absolute ethanol was added to 180 μL of DPPH solution (0.2 mM, in absolute ethanol). After mixing and incubation for 30 min, the absorbance was measured at 518 nm with an ELISA reader (TECAN, Salzburg, Austria). The free radical scavenging activity was calculated as follows: inhibition rate (%) = (1 − (sample O.D./control O.D.)) × 100. IC_50_ values were defined as the concentration at which 50% of DPPH free radicals were scavenged. The positive control was l-ascorbic acid.

#### 3.5.2. Measurement of NBT/Superoxide Scavenging Activity

A reaction mixture with a final volume of 632 μL was prepared with 50 mM phosphate buffer (pH 7.5) containing EDTA (0.05 mM), hypoxanthine (0.2 mM), 63 μL NBT (1 mM) (Sigma-Aldrich), 63 μL of aqueous solution or extract (distilled water for the control), and 63 μL of xanthine oxidase (1.2 U/μL) (Sigma-Aldrich). The xanthine oxidase was added last. For each sample, a blank was also included. The rate of NBT reduction was determined by sequential spectrophotometric determination of absorbance at 590 nm. The solutions were prepared daily and kept shielded from light. The results are expressed as the percentage inhibition of NBT reduction with respect to the reaction mixture without sample (buffer only). Superoxide anion scavenging activities were calculated as ((1 − (sample O.D. − blank O.D.)/(control O.D. − blank O.D.)) × 100) and were expressed as IC_50_ values, defined as the concentration at which 50% of NBT/superoxide anions were scavenged. Allopurinol was used as the positive control.

### 3.6. Measurement of Inhibition of NO Production

Raw 264.7 macrophage cells were cultured in a 96-well plate and incubated for 3 h at 37 °C in a humidified atmosphere (5% CO_2_). The cells were then incubated in a medium containing 0.1 ug/mL lipopolysaccharide (LPS; Sigma-Aldrich) and samples. After incubating for an additional 24 h, the NO content was analyzed by Griess assay. Griess reagent (0.1% naphthylethylenediamine dihydrochloride and 1% sulfanilamide in 5% H3PO4 solution; Sigma-Aldrich) was added to the supernatant from cells treated with each sample. *N*^G^-Monomethyl-l-arginine (l-NMMA) was used as a positive control. NO content was then read at 540 nm against a standard sodium nitrite curve [48]. Inhibitory activity against NO production was calculated as inhibition rate (%) = (1 − (sample O.D. − blank O.D.)/(control O.D. − blank O.D.)) × 100 and IC_50_ values were defined as the concentration that inhibited 50% of NO production.

### 3.7. Measurement of Inhibitory Activity on Cytokine Production

The concentration of cytokines (IL-1β, IL-6, TNF-α (eBioscience, San Diego, CA, USA) in culture supernatants was measured by enzyme-linked immunosorbent assay (ELISA). Cytokine content was quantified by measuring the absorbance at 405 nm with an ELISA reader (TECAN). The amount of cytokines production was calculated using a standard calibration curve. After THP-1 cells were exposed by LPS, levels of the cytokines were measured for inhibitory effect of all compounds (**1**–**7**) and positive control (EGCG) at 50 μM.

### 3.8. Preparation of Liver Microsomes

Liver microsomes were prepared from male rats. Two mature Sprague-Dawley male rats were sacrificed and the livers were removed and minced in a beaker with a pair of scissors. The minced tissue was homogenized in three tissue volumes of medium A (0.32 M sucrose, 1 mM dithiothreitol, and 20 mM sodium phosphate, pH 6.5) and the homogenate was centrifuged at 10,000× *g* for 10 min. The resulting pellet was washed with two pellet volumes of medium A. The combined supernatant from the two centrifugations was suspended in 4 mL medium A, and dispersion of microsomes was achieved using a syringe with 18 G, 23 G, and 25 G needles in succession. The microsome suspension was divided into aliquots and stored at −80 °C. The microsomes were diluted with medium just before use.

### 3.9. Measurement of Inhibitory Activity Against 5α-reductase

5α-Reductase from rat liver microsomes was incubated with 400 μL phosphate buffer (pH 6.5) on Intact group and 200 μL phosphate buffer on Normal group (negative control), 50 μL testosterone as substrate (100 μg/mL), 200 μL (1 mM) finasteride on positive control group (finasteride) or 200 μL (1 mM) of compounds (**1**–**7**), and 20 μL NADPH (0.8 mg/mL). Microsome enzyme (5α-reductase) isolated from rat liver was added to all groups except for the intact group with 200 μL. The reaction was terminated by 0.5 mL dichloromethane was added for every group. The amount of testosterone as substrate for 5α-reductase was measured by HPLC. The injection volume was 20 μL and elution was performed at a flow rate of 1 mL/min using a binary gradient of H_2_O (A) and acetonitrile (ACN) (B). The quantification wavelength of these chromatograms was set at 242 nm, which was optimized for testosterone. The data were integrated using the Empower software system (Waters, coastal, CT, USA).

### 3.10. Macrophage Differentiation and Stimulation

The mature macrophage-like state was induced by treating THP-1 monocytes (10^5^ cells/mL) for 48 h with 10 nmol 12-*O*-tetradecanoylphorbol-13-acetate (TPA; Sigma-Aldrich) in 24-well cell culture plates with 1 mL cell suspension in each well. Differentiated plastic-adherent cells were washed once with PBS and provided with fresh RPMI 1640 medium (Sigma-Aldrich) containing 10% FBS and 100 IU/mL penicillin G (Gibco BRL). Differentiated THP-1 cells were treated with test samples and 0.1 μg/mL LPS (Sigma-Aldrich) and incubated for 1 h at 37 °C in a humidified atmosphere (5% CO_2_). After further incubation for 1 day, supernatant was transferred to Eppendorf tubes for cytokine assays.

### 3.11. Statistical Analysis

Values were analyzed by one-way analysis of variance (ANOVA) followed by Student-Newman-Keuls (S-N-K) test and one to one confrontation test that figure out *t*-value, *p*-value with the Statistical Package for the Social Sciences (SPSS) software pack (IBM, Armonk, NY, USA).

## 4. Conclusions

Repeated chromatographic fractionation of an 80% acetone extract of the leaves of AR yielded seven phenolic compounds **1**–**7**. The caffeoyl derivatives showed higher anti-oxidative activities than flavones; in particular, caffeic acid (**1**) and grandifloroside (**7**) displayed potent anti-inflammatory activity in the NO production assay. Caffeoyl derivatives **1**–**3** displayed more potent inhibitory activities on productions of pro-inflammatory cytokines than flavones. Also grandifloroside (**7**) showed remarkable inhibitory effect on 5α-reductase activity. These results suggest that the leaves of AR and its caffeoyl derivatives and caffeoyl secoiridoid, especially grandifloroside (**7**) (the first isolated secoiridoid from this plant) might be developed as anti-oxidant, anti-inflammatory, and potential therapeutic agents for the treatment of BPH.

## Figures and Tables

**Table 1:** IC_50_ values of anti-oxidative and anti-inflammatory activities of compounds from AR.

Compounds	IC_50_ (μM)		
	DPPH Scavenging Radical Activity	NBT Superoxide Scavenging Activity	NO Inhibitory Production Activity
**1**	33.97 ± 1.60 ^c^	8.52 ± 1.08 ^b^	23.98 ± 1.52 ^a^
**2**	40.30 ± 2.22 ^d^	9.75 ± 1.44 ^b^	57.01 ±1.76 ^d^
**3**	24.39 ± 1.80 ^a^	7.08 ± 0.91 ^b^	91.77 ± 2.01 ^e^
**4**	29.62± 0.22 ^b^	15.05 ± 1.31 ^c^	48.63 ± 2.01 ^c^
**5**	34.31 ± 1.50 ^c^	42.83 ± 1.81 ^d^	46.69 ± 1.15 ^c^
**6**	> 100 ^e^	>100 ^e^	45.35 ± 2.32 ^c^
**7**	27.06 ± 0.24 ^a^	9.24 ± 0.43 ^b^	30.12 ±1.02 ^b^
Ascorbic Acid	23.88 ± 0.76 ^a^	-	-
Allopurinol	-	4.57 ± 0.73 ^a^	-
l-NMMA	-	-	29.12 ± 1.98 ^b^

Values are presented as the mean ± SD (*n* = 3); Values bearing different superscripts ^a^, ^b^, ^c^, ^d^, ^e^ in the same column are significantly different. *p* < 0.05.

**Table 2:** IC_50_ values of anti-oxidative and anti-inflammatory activities of fractions from AR.

Fractions	IC_50_ (μg/mL)		
	DPPH Scavenging Radical Activity	NBT Superoxide Scavenging Activity	NO Inhibitory Production Activity
Extract	56.40 ± 2.78 ^f^	85.39 ± 1.44 ^b^	18.01 ± 1.21 ^b^
Fr.1	> 100 ^h^	>100 ^c^	> 100 ^e^
Fr.2	> 100 ^h^	>100 ^c^	> 100 ^e^
Fr.3	> 100 ^h^	>100 ^c^	> 100 ^e^
Fr.4	73.37 ± 0.59 ^g^	83.70 ± 1.77 ^a^	77.32 ± 1.07 ^d^
Fr.5	15.20 ± 1.15 ^a^	>100 ^c^	17.34 ± 0.82 ^b^
Fr.6	58.26 ± 2.96 ^e^	87.64 ± 1.09 ^b^	21.63 ± 0.30 ^c^
Fr.7	19.59 ± 2.56 ^b^	>100 ^c^	16.38 ± 2.86 ^b^
Fr.8	31.92 ± 3.03 ^d^	>100 ^c^	> 100 ^e^
Fr.9	23.26 ± 3.49 ^c^	>100 ^c^	> 100 ^e^
Fr.10	35.04 ± 1.29 ^d^	-	> 100 ^e^
Ascorbic Acid	23.40 ± 0.49 ^c^	-	-
Allopurinol	-	89.44 ± 0.74 ^b^	-
l-NMMA	-	-	12.5< ^a^

Values are presented as the mean ± SD (*n* = 3); Values bearing different superscripts ^a^, ^b^, ^c^, ^d^, ^e^ in the same column are significantly different. *p* < 0.05.
